# A Collection of Benchmark Data Sets for Knowledge Graph-based Similarity in the Biomedical Domain

**DOI:** 10.1093/database/baaa078

**Published:** 2020-11-11

**Authors:** Carlota Cardoso, Rita T Sousa, Sebastian Köhler, Catia Pesquita

**Affiliations:** Departamento de informática, LASIGE Faculdade de Ciências da Universidade de Lisboa, 1749 - 016 Lisboa, Portugal; Departamento de informática, LASIGE Faculdade de Ciências da Universidade de Lisboa, 1749 - 016 Lisboa, Portugal; Ada Health GmbH Karl-Liebknecht-Str. 1. 10178 Berlin; Departamento de informática, LASIGE Faculdade de Ciências da Universidade de Lisboa, 1749 - 016 Lisboa, Portugal

## Abstract

The ability to compare entities within a knowledge graph is a cornerstone technique for several applications, ranging from the integration of heterogeneous data to machine learning. It is of particular importance in the biomedical domain, where semantic similarity can be applied to the prediction of protein–protein interactions, associations between diseases and genes, cellular localization of proteins, among others. In recent years, several knowledge graph-based semantic similarity measures have been developed, but building a gold standard data set to support their evaluation is non-trivial.

We present a collection of 21 benchmark data sets that aim at circumventing the difficulties in building benchmarks for large biomedical knowledge graphs by exploiting proxies for biomedical entity similarity. These data sets include data from two successful biomedical ontologies, Gene Ontology and Human Phenotype Ontology, and explore proxy similarities calculated based on protein sequence similarity, protein family similarity, protein–protein interactions and phenotype-based gene similarity. Data sets have varying sizes and cover four different species at different levels of annotation completion. For each data set, we also provide semantic similarity computations with state-of-the-art representative measures.

**Database URL**: https://github.com/liseda-lab/kgsim-benchmark.

## Introduction

The growing size of data produced by nearly all domains of human endeavour brought with it new challenges in handling the size, the complexity and the diversity of data. One of the domains where this data deluge has altered nearly every aspect of its workings is the life sciences. High-throughput techniques in genomics and proteomics produce large amounts of data about the function, regulation and interaction of genes and proteins, and their integration with clinical research has helped link thousands of genes to related diseases.

The size of the data, but also the underlying complexity in describing it, was a strong motivator for the adoption of ontologies by the biomedical community. Since the early 2000s, biomedical ontologies have been increasingly used to annotate data, which has resulted in a proliferation of ontologies (more than 800 currently stored in BioPortal; https://bioportal.bioontology.org/) and ontology annotated data sets, many available as linked open data (e.g. Bio2RDF; https://bio2rdf.org/). The creation of these resources, as is also the case of more general-purpose knowledge graphs (KGs) such as DBpedia (1) or Yago, was the result of tremendous efforts by the scientific community to provide a way to make data understandable by both humans and machines.

The ability to describe complex entities by linking them to the ontology concepts that describe them supports the computation of the similarity between entities with algorithms that explore ontology features (2). Several tasks can be supported by these semantic similarity metrics, such as integration of heterogeneous data, entity matching, comparison and clustering and generation of recommendations. In fact, computing similarity between instances is an integral part of many machine learning techniques, both supervised and unsupervised. In the biomedical domain, the ability to compare entities, such as genes, cells, organisms, populations, species, and finding their similarities and differences is essential to support scientific inquiry. While comparing the sequences of two genes or the structures of two proteins can be achieved directly, because both have objective representations and measurable properties, ontologies provide mechanisms of objective representation that support measurement of more complex aspects, such as function.

Biomedical semantic similarity has been successfully applied to such diverse tasks as protein–protein interaction (PPI) prediction (3–5), prediction of disease-associated genes (3) or drug-target interaction prediction (6). It is worth noting that in these applications, similarity is not used to detect identity, but rather to predict the likelihood of a given entity exhibiting a given property.

There are several measures available (2, 7), each with their distinguishing characteristics. Given the variety of approaches and measures for semantic similarity, it is fundamental to determine the best measure for each application scenario. However, there is no gold standard for similarity between complex biomedical entities, and a manual assessment of similarity by domain experts is unfeasible, not only due to the size of the data but also because each expert is inherently biased towards a viewpoint of the domain or a particular use case. Furthermore, each of the existing measures formalizes the notion of similarity in a slightly different way, and for that reason, it is not possible to define what the best semantic similarity measure would be, since it becomes a subjective decision.

One possible solution is to compare semantic similarity measures to other measures or proxies of similarity. In the biomedical domain, entities can be compared through different lenses. For instance, we can compare two genes via their sequence similarity, two proteins via their structural similarity or two diseases by the metabolic pathways they affect. These similarities do not provide the broad-spectrum comparison that semantic similarity supports, but they are known to relate to relevant characteristics of the underlying entities. As such, these similarity proxies can be compared to the semantic similarity to help understand how well a semantic similarity approach captures entity similarity.

We present a collection of 21 benchmark data sets that aim at circumventing the difficulties in building benchmarks for large biomedical KGs by exploiting proxies for biomedical entity similarity. These data sets are grouped according to the KGs and proxy measures they are based on: (1) ‘Protein Family Similarity’, based on the Gene Ontology (GO) (8); (2) ‘Protein–Protein Interaction’, also based on the GO and (3) ‘Phenotype-based Gene Similarity’, based on the Human Phenotype Ontology (HPO) (9). The data sets vary in size, both in terms of individual entities and entity pairs, from a few hundred to over hundred thousand pairs. For each data set, we also provide semantic similarity computations with four state-of-the-art representative measures.

## Biomedical KGs

Ontologies are structured representations of a domain of human knowledge that are made up of classes, descriptors of the features of the entities in that domain and a set of relations between these classes (10). Ontologies can be used to describe real-world entities through the process of semantic annotation: entities are linked with the ontology classes most fitting to describe them. A set of entities annotated with a given ontology constitute a KG. With this structured representation of reality, it is possible to computationally reason over the entities, streamlining a process that would be much more expensive and time consuming if done by humans.

**Figure 1.**
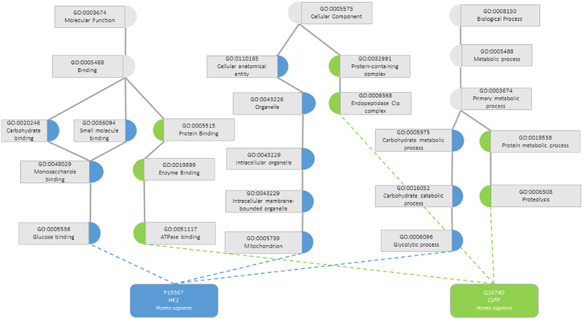
Semantic similarity in the Gene Ontology between proteins P19367 and Q16740. Blue circles are classes that only annotate protein P19367, the green circles are classes that only annotate protein Q16740 and grey circles are classes that annotate both proteins.

One task that was made possible with the development of KGs was the calculation of semantic similarity between the entities. A semantic similarity measure is a function that, given two ontology classes or two sets of classes describing two individuals, returns a numerical value reflecting the closeness in meaning between them (7). Figure 1 illustrates how two proteins are represented by their GO classes and how they can be compared through semantic similarity measures. An accurate assessment of the similarity between a pair of entities depends on how well they are annotated, both regarding breadth (i.e. including annotations for all aspects of the entity that can be described within the ontology domain) and depth of annotations (i.e. choosing the most specific classes of the ontology that best describe the entity). If two entities are shallowly annotated with the same non-specific classes, they can be rendered similar but be, in fact, very dissimilar, which would impact semantic similarity performance (11, 12).

The GO is the most successful case of the use of an ontology in the biomedical domain. The GO is a directed acyclic graph that covers three distinct aspects of gene product’s role: Molecular Function (its activity at the molecular level), Cellular Component (the location of its activity relative to biological structures) and Biological Process (a larger biological program in which its molecular function is utilized) (8). The GO Annotation is a project that aims to provide assignments of GO classes to proteins of any species, i.e. annotations (13). The annotations provide a standardized way to describe proteins, which makes them directly comparable with semantic similarity metrics.

GO-based semantic similarity is the main research focus of semantic similarity in molecular biology, with several GO-based semantic similarity measures being developed through the years (7, 14, 15). Overall, semantic similarity in the GO has been applied mainly for validating and predicting functions and interactions, and for analysing transcriptomics and proteomics data (7). In predicting and validating the function of gene products, these measures can be combined with other similarity metrics, as structural similarity (16) or sequence similarity (17, 18) for better results. In PPI prediction and validation, similarly, semantic similarity can be the sole approach (5, 19) or be used to improve already existing techniques (20). Finally, the role of semantic similarity in the analysis of transcriptomics and proteomics data is mainly the improvement of clustering of co-expressed gene products (21–23).

Another example of an ontology of interest in the biomedical domain is the HPO. The HPO contains about 15 000 concepts describing phenotypic abnormalities found in human hereditary diseases and is divided into independent subontologies that cover different categories: ‘Phenotypic abnormality’, ‘Mode of inheritance’, ‘Clinical course’, ‘Clinical modifier’ and ‘Frequency’. The HPO has been used to integrate sequencing data from multiple biotech centres to identify patients with mutations in the same gene and comparable phenotypes, to record detailed clinical phenotypes of patients with rare inherited disorders, to annotate clinical cases with standard phenotype variants in order to cluster phenotypically overlapping patients and finally to increase interoperability between clinical laboratories (9, 24).

Semantic similarity using the HPO has many applications. Different semantic similarity-based techniques can be used to rank diseases annotated with HPO based on how similar they are to several queries of HPO classes (9, 25). This method allows for the clinical diagnosis of patients by finding the most similar disease to their set of symptoms Similarly, Masino *et al*. (26) use semantic similarity and the HPO to predict the disease-causing gene in patients. Some HPO-based semantic similarity measures have been developed throughout the years (9, 27, 28).

## Related work

Building a gold standard data set to support semantic similarity evaluation is not trivial. Accomplishing this manually is extremely time-consuming, and existing manual gold standards are minimal when compared to the size of the ontologies they correspond to. For instance, Pedersen *et al*. (29) created a set of only 30 term pairs extracted from Unified Medical Language System Metathesaurus. The Unified Medical Language System (30) contains over 1 million biomedical concepts and 5 million concept names, originating from more than 100 incorporated controlled vocabularies and classification systems.

To mitigate this challenge, some semantic web-related applications have turned to crowdsourcing (e.g. in ontology matching (31) and in the verification of relations (32)) which brings with it a series of new challenges.

The evaluation task can be inherently biased towards a particular viewpoint of the domain or a particular use case. This can be of extreme relevance in biomedical semantic similarity, where two genes may be deemed similar across many different axes: molecular function, expression, cellular localization, disease involvement, etc. The evaluation task success is also highly dependent on the ability to provide crowdsourced workers with enough information to make a decision.

In previous work, we have developed Collaborative Evaluation of Semantic Similarity Measures (CESSM) (33). This tool enables the comparison of new GO-based semantic similarity measures against previously published ones considering their relation to sequence, Pfam (34) and Enzyme Commission (EC) (35) number similarity. CESSM was released in 2009 and updated in 2014, and since then it has been widely used by the community, being adopted to evaluate over 25 novel semantic similarity measures developed through different methods, with more recent ones focusing on common information content (IC)-based metrics (36) but also based on vector representations/graph embeddings (37). CESSM was built as a web-based tool to support the automatic comparison against the benchmark data. Over time, we identified some limitations of its use: users looking to perform iterative evaluations were limited by access through a graphical user interface; users were unable to calculate other metrics of performance not supported by the tool; users were limited to a single ontology (GO) and a single functional perspective given by the Pfam and EC proxies which focus on protein function similarity. Despite its limitations, CESSM’s methodology for evaluation of semantic similarity measures showed effectiveness and its data set was successfully used for other tasks than the evaluation techniques of semantic similarity measures supported by the tool (3).

Other data sets have also been used to evaluate KG-based semantic similarity. MateTee, a KG-based semantic similarity metric (38) was evaluated both with CESSM and with a gold standard based on DBpedia entities of the type Person. AnnSim (39) was also evaluated on CESSM, but it also included additional evaluations based on drug–target interaction prediction using other data sets. However, the drug–target interaction data sets only provide the proxy-based similarity (in this case, a link between drug and target) and do not provide the necessary KG annotations for each data item. These are likely to change over time, so a fair and unbiased comparison between different tools would need to be run with the exact versions of the data.

Finally, there are related contributions in the area of benchmark data for several machine learning tasks in KGs, such as link prediction (40, 41) and classification (42). The Open Graph Benchmark includes a biomedical KG but focuses only on link property prediction (43). KG-based semantic similarity can be applied in these contexts, but these benchmark data sets do not support a direct evaluation of semantic similarity measures.

## Materials and methods

### Ontologies and KG data

The benchmark data sets in this collection are grouped according to the KGs and proxy measures they are based on: (1) ‘Protein Family Similarity’, based on the GO, (2) ‘Protein–Protein Interaction’, also based on the GO and (3) ‘Phenotype-based Gene Similarity’, based on the HPO.

The protein benchmark data sets are constituted by proteins, identified by their UniProt Accession Numbers. Each protein is annotated with classes (also commonly referred to as terms) from the GO. There are species-specific data sets (*Drosophila melanogaster, Escherichia coli, Homo sapiens* and *Saccharomyces cerevisiae*) and a data set that groups these species (‘All’). In September 2019, 12 490 *D. melanogaster*, 5341 *E. coli*, 19 464 *H. sapiens* and 6048 *S. cerevisiae* proteins were annotated with GO.

The gene benchmark data set is constituted by genes, identified by their Entrez Gene Code and annotated with classes from the HPO. These annotations link the genes with the HPO classes which best describe the disease in which the genes have been shown to play a role. The benchmark KG contains 4293 human genes and their annotations to the HPO (dated November 2019).

### KG-based semantic similarity

The approaches used to quantify semantic similarity can be distinguished based on which entities they intend to compare: there are approaches for comparing two classes within an ontology and approaches for comparing two individuals each linked to their own set of classes. When comparing classes, these measures can be node-based, meaning they explore the properties of each class involved, or edge-based, which rely on the distance between the classes. However, edge-based measures are based on the assumption that the nodes and the edges are uniformly distributed through the ontology, which is mostly not true for biomedical ontologies, making node-based measures more reliable (7). These typically rely on the IC of a class, which is a measure of how informative, or rather, specific a class is. The IC can be calculated based on the graph structure (intrinsic approach) or based on the usage of the class in annotating entities in a corpus (extrinsic approach). In this work, we focus on two IC measures, presented below, which are representative of each type.

${\rm{I}}{{\rm{C}}_{{\rm{Seco}}}} $ (44) is a structural IC based on the number of direct and indirect children of a class ${\rm{c}}$ and is given by

$$\begin{equation*}{\rm{I}}{{\rm{C}}_{{\rm{Seco}}}}\left( {\rm{c}} \right) = 1 - {{\log \left[ {{\rm{hypo}}\left( {\rm{c}} \right) + 1} \right]} \over {{\rm{log }}\left[ {maxnodes} \right]}}\end{equation*}$$

where ${\rm{hypo}}\left( {\rm{c}} \right)$ is the number of direct and indirect children from class ${\rm{c}}$ (including class ${\rm{c}}$) and ${\rm{maxnodes}}$ is the total number of classes in the ontology.

${\rm{I}}{{\rm{C}}_{{\rm{Resnik}}}}$ (45) is a corpus-based approach and based on the number of entities annotated with class c in a KG:

$$\begin{equation*}{\rm{I}}{{\rm{C}}_{{\rm{Resnik}}}}\left( {\rm{c}} \right) = - \log {\rm{p}}\left( {\rm{c}} \right)\end{equation*}$$

where ${\rm{p}}\left( {\rm{c}} \right)$ is the probability of annotation in the corpus.

The normalized version of ${\rm{I}}{{\rm{C}}_{{\rm{Resnik}}}}$ is given by

$$\begin{equation*}{\rm{I}}{{\rm{C}}_{{\rm{norm}}}}\left( {\rm{c}} \right) = {{{\rm{I}}{{\rm{C}}_{{\rm{Resnik}}}}\left( {\rm{c}} \right)} \over {\log {\rm{N}}}}\end{equation*}$$

with ${\rm{N}}$ being the total number of annotations.

To calculate semantic similarity for two individuals, each described with a set of classes, both pairwise and groupwise approaches can be used. Pairwise approaches assess the similarity between two individuals by combining the semantic similarities between their annotating classes. Groupwise approaches employ vector or graph-based measures that process annotations taken together as a whole. One semantic similarity measure representative of each approach is used to build the benchmark data sets.

‘Best Match Average’ (BMA) is a pairwise approach based on the pairwise measure in which the similarity between two classes corresponds to the IC of their most informative common ancestor (45). In BMA, only the best-matching class for each class in each set of classes describing the individuals (i.e. the most similar) is considered to calculate the pairwise similarity, given by

$$\begin{equation*}{\rm{BMA}}\left( {{\rm{A}},{\rm{B}}} \right) = {{\mathop \sum \nolimits_{{{\rm{c}}_1} \in {{\rm{C}}_{\rm{A}}}} {\rm{sim}}\left( {{{\rm{c}}_1},{{\rm{c}}_2}} \right)} \over {2\left| {{C_A}} \right|}} + {{\mathop \sum \nolimits_{{{\rm{c}}_2} \in {{\rm{C}}_{\rm{B}}}} {\rm{sim}}\left( {{{\rm{c}}_1},{{\rm{c}}_2}} \right)} \over {2\left| {{C_B}} \right|}}\end{equation*}$$

where ${\rm{A}}$ and ${\rm{B}}$ are entities, ${\rm{C}}$ is the set of classes ${\rm{c}}$ each entity is described with and ${\rm{sim}}\left( {{{\rm{c}}_1},{{\rm{c}}_2}} \right)$ and ${\rm{sim}}\left( {{{\rm{c}}_2},{{\rm{c}}_1}} \right)$ are the highest similarity values found for class ${{\rm{c}}_1},{{\rm{c}}_2}.$ The similarity between two classes can be found using Resnik’s similarity (45):

$$\begin{equation*}{\rm{sim}}\left( {{{\rm{c}}_1},{{\rm{c}}_2}} \right) = {\rm{max}}\left[ {{\rm{IC}}\left( {\rm{c}} \right)} \right]:{\rm{c}} \in {\rm{A}}\left( {{{\rm{c}}_1}} \right) \cap {\rm{A}}\left( {{{\rm{c}}_2}} \right)\end{equation*}$$

where ${\rm{A}}\left( {{{\rm{c}}_{\rm{i}}}} \right)$ is the set of ancestors of ${{\rm{c}}_{\rm{i}}}$.

${\rm{SimGIC}}$ (46) is a groupwise approach which resorts to the Jaccard similarity, in which each class ${\rm{c}}$ is weighted by its IC. It is given by

$$\begin{equation*}{\rm{simGIC}}\left( {{\rm{A}},{\rm{B}}} \right) = {{\mathop \sum \nolimits_{{\rm{c}} \in {{\rm{C}}_{\rm{A}}}\cap {{\rm{C}}_{\rm{B}}}} {\rm{IC}}\left( {\rm{c}} \right)} \over {\mathop \sum \nolimits_{{\rm{c}} \in {{\rm{C}}_{\rm{A}}} \cup {{\rm{C}}_{\rm{B}}}} {\rm{IC}}\left( {\rm{c}} \right)}}\end{equation*}$$

where ${\rm{A}}$ and ${\rm{B}}$ are entities, ${\rm{C}}$ is the set of classes ${\rm{c}}$ each entity is annotated with.

To produce data sets that are representative in terms of semantic similarity measures, we combined both IC measures with each entity similarity measure to arrive at four state-of-the-art semantic similarity measures employed in the benchmark: $BM{A_{Resnik}},BM{A_{Seco}},simGI{C_{Resnik}}$ and $simGI{C_{Seco}}.$

### Building the benchmark data sets

**Figure 2.**
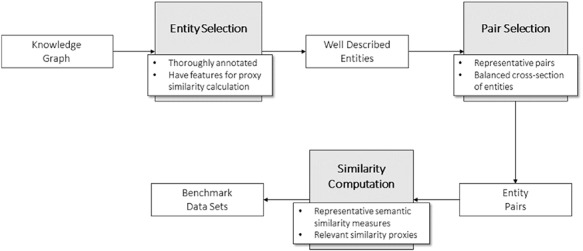
General methodology for the development of the data sets.

To build these benchmark data sets, we developed a general methodology (Figure 2) divided in three steps. The first step consists of selecting the entities of the KG that will make up the pairs in the data sets. These entities should be well characterized in the context of the ontology to avoid the shallow annotation bias and have enough information to compute proxy similarity between them.

Next step is generating the pairs of entities. In doing so, we tried to guarantee a wide range of similarity among the pairs of entities, from null to total identity to ensure representativeness in the pairs of entities (see ‘Technical validation’).

Finally, having selected the pairs of entities for the data set, the two types of similarity measures need to be calculated: semantic similarity (see KG-based semantic similarity) and similarity proxies that are relevant for the type of entities and data sets at hand.

### Protein benchmark data sets

We have created two types of protein benchmark data sets, one based on protein family similarity and another based on PPIs. In building them, the first step is to select the proteins that will constitute them. This includes filtering proteins without sufficient GO annotations, as well as proteins for which the necessary information to compute proxy similarity is not available.

### Protein selection based on GO annotations

In GO semantic similarity, not only is the depth of the GO terms an important feature, as previously exposed, as is the breadth of annotations within the three GO aspects (Molecular Function, Cellular Component, and Biological Process) since measures may wish to handle them differently. To tackle these issues, we created two different types of data sets according to the following criteria:

‘One aspect’: The proteins must have at least one annotation in each GO aspect, and in at least one aspect, there should be at least one leaf-class annotation.

‘All aspects’: The proteins must have at least one annotation in each GO aspect, and in each aspect, there should be at least one leaf-class annotation.

This ensures that all proteins are sufficiently annotated to support semantic similarity calculations in either one or all aspects of GO. It also results in all proteins in the ‘All aspects’ data set being included in the ‘One aspect’ as well.

### Measuring protein similarity

We employ three proxies of protein similarity based on their biological properties: sequence similarity, functional domain similarity and PPIs.

***Sequence similarity***: Protein sequence similarity measures the relationship between two sequences, and it establishes the likelihood for sequence homology. Sequence similarity ($si{m_{Seq}}$) will be calculated through the relative reciprocal BLAST score (46):

$$\begin{equation*}si{m_{Seq}}\left( {{\rm{A}},{\rm{B}}} \right) = {{{\rm{BLAS}}{{\rm{T}}_{{\rm{bitscore}}}}\left( {{\rm{A}},{\rm{B}}} \right) + {\rm{BLAS}}{{\rm{T}}_{{\rm{bitscore}}}}\left( {{\rm{B}},{\rm{A}}} \right)} \over {{\rm{BLAS}}{{\rm{T}}_{{\rm{bitscore}}}}\left( {{\rm{A}},{\rm{A}}} \right) + {\rm{BLAS}}{{\rm{T}}_{{\rm{bitscore}}}}\left( {{\rm{B}},{\rm{B}}} \right)}}\end{equation*}$$

where ${\rm{A}}$ and ${\rm{B}}$ are two proteins.

The relationship between sequence similarity and sema-ntic similarity is non-linear (46) but becomes more relevant the higher the sequence similarity is (47). While sequence similarity can be used to evaluate the performance of a semantic similarity measure, it should not be the sole evaluator in this task.

***Pfam similarity:*** Protein family similarity is computed by comparing the functional regions (commonly termed domains) that exist in each protein sequence using the equation below. Protein functional domains are extracted from the Pfam references contained in the UniProt database. Pfam similarity ($si{m_{Pfam}}$) is calculated as a Jaccard similarity, using the ratio between the number of families common to proteins A and B and the total number of distinct families through proteins A and B:

$$\begin{equation*}{\rm{si}}{{\rm{m}}_{{\rm{Pfam}}}}\left( {{\rm{A}},{\rm{B}}} \right) = {{\left| {{{\rm{f}}_A} \cap {f_B}} \right|} \over {\left| {{{\rm{f}}_A} \cup {f_B}} \right|}}\end{equation*}$$

where ${\rm{A}}$ and ${\rm{B}}$ are two proteins with the set of families ${f_A}$ and ${f_B}$, respectively.

The more functional domains two proteins share, the more likely will be that their semantic similarity in the GO is high, especially in the Molecular Function aspect since these domains are usually responsible by assigning functions to proteins (34).

***Protein–protein interactions:*** In these data sets, PPI has a binary representation: 1 if the proteins interact, 0 otherwise. The data sets we employed to extract interacting and non-interacting pairs correspond to a set of well-known benchmarks for PPIs covering the four species (presented in Table 1). We consider two proteins to be similar if they interact. PPIs have some correlation to semantic similarity in the GO: if two proteins are co-localized in the cell and involved in the same large-scale process, they are most likely to interact and will share some GO terms in the Biological Process and Cellular Component aspects. Both Sousa *et al*. (48) and Maetschke *et al*. (4) show this to be true, whose PPI predicting approaches demonstrate higher predictive power when using terms from these two aspects. However, two proteins can be very similar through different lenses (e.g. having high sequence, semantic or Pfam similarity) but not interact.

**Table 1.** PPI benchmark data sets, with original publication reference and protein’s species

Data set	Species
STRING-SC (4)	*S. cerevisiae*
STRING-HS (4)	*H. sapiens*
STRING-EC (4)	*E. coli*
STRING-DM (4)	*D. melanogaster*
DIP-HS (5)	*H. sapiens*
BIND-SC (53)	*S. cerevisiae*
DIP/MIPS-SC (53)	*S. cerevisiae*
GRID/HPRD-BAL-HS (51)	*H. sapiens*
GRID/HPRD-UNBAL-HS (51)	*H. sapiens*

The semantic similarity measures employed for these data sets are the previously presented ones.

### Protein family similarity data set

In the Protein family similarity data sets, two similarity proxies are employed: sequence and protein family similarity. Protein family similarity is computed using equation for $si{m_{Pfam}}$ and Pfam assignments to proteins. Thus, proteins in these data sets should also have at least one domain identified in the Pfam database, which further filters down the number of proteins in this data set. After selecting the eligible proteins, pairs were randomly generated. This, of course, results in a very large number of proteins having zero $si{m_{Pfam}}$, so we randomly filtered the pairs to ensure the same number of pairs with zero, partial and total $si{m_{Pfam}}$.

### PPI data set

In the PPI data sets, two similarity proxies are employed: sequence and PPIs. Although sequence similarity is not as highly correlated to protein interactions as it is with molecular functions, it has been successfully used to predict PPIs. By combining the initial protein selection criteria with the PPI benchmark data sets, we filtered the pairs down to only include pairs where both proteins met the set criteria. Then, the pairs of proteins were grouped by species, excluding all existing duplicates, and the data sets were filtered to ensure that the number of pairs of interacting and non-interacting proteins was the same in each data set.

### Gene-phenotypes benchmark data set

The gene-phenotypes benchmark data set is constituted by genes, identified by their official gene symbol and annotated with the HPO. For this data set, all human genes meeting the following criteria were considered:

The gene must be described with at least three distinct HPO classes in the subontology ‘Phenotypic Abnormality’ (i.e. not ancestors/descendants of each other);
The gene must have a link with at least one phenotype in any ‘Phenotypic Series’ (PS), and the mechanism behind that link must be known.

This ensures that the benchmark is composed of sufficiently annotated genes, for which the proxy similarity is possible to compute. These criteria take into consideration that disease-causing genes are typically associated with several phenotypic abnormalities and that HPO annotations are not as frequent at the leaf level.

### Measuring genes’ phenotypes similarity

The proxy similarity is based on OMIM’s PS (49), which are groups of identical or similar phenotypes and their associated genes. To date, OMIM has 464 different PS composed of 3777 phenotypes. Information on how genes relate to PS was retrieved from OMIM (https://www.omim.org/phenotypicSeriesTitles/all). PS similarity ($si{m_{PS}}$) is defined as a Jaccard similarity, the ratio between the number of PS common to genes A and B and the total number of distinct PS through genes A and B:

$$\begin{equation*}{\rm{si}}{{\rm{m}}_{{\rm{PS}}}}\left( {{\rm{A}},{\rm{B}}} \right) = {{\left| {{\rm{P}}{{\rm{S}}_A} \cap P{S_B}} \right|} \over {\left| {{\rm{P}}{{\rm{S}}_A} \cup P{S_B}} \right|}}\end{equation*}$$

where ${\rm{A}}$ and ${\rm{B}}$ are two genes with the set of Phenotypic Series $P{S_A}$ and $P{S_B}$, respectively.

Similarly to $si{m_{Pfam}}$ in the protein family protein data sets, $si{m_{PS}}$ correlates to semantic similarity because the more PS two genes are associated with, the more likely it is that they share HPO classes in the ‘Phenotypic Abnormality’ subontology, since PS are a set of similar phenotypes.

**Figure 3.**
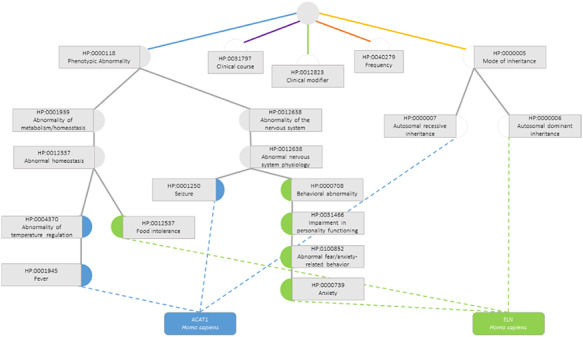
Semantic similarity inside the Phenotypic Abnormality sub-ontology in the Human Phenotype Ontology between the genes ACAT1 and ELN. Blue circles are classes that only annotate gene ACAT1, green circles are terms that only annotate gene ELN, grey circles are classes that annotate both genes and white circles are classes whose annotations are discarded due to not belonging to the Phenotypic Abnormality sub-ontology.

The semantic similarity measures employed for this data set are the previously presented ones, calculated considering only the terms in the subontology ‘Phenotypic Abnormality’ (Figure 3), since these are the ones that truly influence the functional similarity of the genes.

### Genes-phenotypes data set

After selecting the eligible genes, pairs of genes were randomly generated. Since most genes share no PS and would have zero $si{m_{PS}}$, we randomly filtered the pairs to ensure the same number of pairs with zero, partial and total $si{m_{PS}}$.

## Results

### Protein benchmark data sets

The proposed methodology was employed to produce the data sets described in Table 2. This resulted in 16 data sets, divided by species, level of annotation completion and similarity proxy, and 4 additional data sets, combining all species’ protein pairs in the same proxy group. Data sets sizes range from as little as 287 different proteins and 200 pairs, to 26 000 proteins and 104 000 pairs. Regarding the PPI data sets, there are small differences between the number of proteins and pairs in the ‘One Aspect’ and ‘All Aspect’ data sets. Given that these data sets are based on benchmark data sets of PPIs, it is likely that the proteins therein are well characterized, particularly in the case of *H. sapiens* data sets. This means that most of the proteins in the original data sets meet the criteria for “All Aspects’, explaining the small difference in the sizes of the data sets.

**Table 2.** Species, number of proteins and pairs, annotation completion and proxy measure they are based on for all protein data sets

	PPI				Protein family			
	One aspect		All aspects		One aspect		All aspects	
Species	Proteins	Pairs	Proteins	Pairs	Proteins	Pairs	Proteins	Pairs
*D. melanogaster*	455	364	287	200	7470	31 350	5300	17 682
*E. coli*	371	734	263	420	1231	3363	724	1332
*H. sapiens*	7093	30 826	6718	29 672	13 246	31 350	11 666	25 527
*S. cerevisiae*	3776	27 898	2888	16 904	4782	38 166	3660	29 265
*All*	11 695	59 822	10 156	47 196	26 729	104 229	21 350	73 806

### Genes-phenotypes benchmark data set

Having followed the proposed methodology, a data set with 2026 distinct human genes and 12 000 pairs was produced.

## Technical validation

Benchmark data sets are key to finding the best performing tools for a specific application. There are a number of requirements for good benchmark data sets, namely relevance, representativeness, non-redundancy, scalability and reusability (50). In the context of these benchmark data sets for semantic similarity measures, this means that the data sets should include data relevant for the biomedical domain, have representative cases in both terms of similarity metrics and their values, or contain both positive and negative examples (e.g. positive and negative interactions between proteins, avoid overlapping of cases among them) to make a comparative study between them more relevant, should support the same study in different sized data sets and are of special utility if they can be used for different purposes. Representativeness is of particular relevance in these data sets, as the data sets should provide a balanced cross-section of biomedical entities.

The collection of 21 benchmark data sets we present aims at supporting the large-scale evaluation of semantic similarity measures based on biomedical KGs. It represents an evolution compared with the previous efforts in this area, both in terms of the size and diversity of the data employed (Table 3). The benchmark data can be used to evaluate the multiple components of a semantic similarity measure, i.e. IC, class-based similarity and instance-based similarity approaches. It can also be used to evaluate the impact of semantic similarity on downstream tasks such as PPI prediction or gene–disease association prediction. In this section, we go over the main features of these data sets, displaying their validity for the proposed application.

**Table 3.** Comparison between both updates of CESSM and the presented resource (KG similarity benchmark data sets)

Resource	CESSM 2009	CESSM 2014	KG similarity benchmark data sets
Entities	1039	1626	30 520
Pairs	13 430	22 302	175 489
Ontologies	GO	GO	GO & HPO
Species	Non-specific	Non-specific	4

### Protein benchmark data sets

**Figure 4.**
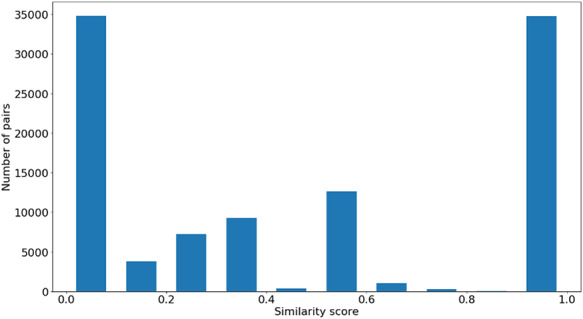
Distribution of ${\rm{si}}{{\rm{m}}_{{\rm{Pfam}}}}$ values across all species’ protein pairs in the protein family data sets.

**Figure 5.**
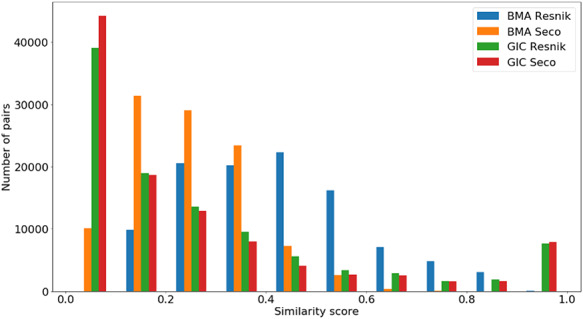
Distribution of all semantic similarity metrics (${\rm{BM}}{{\rm{A}}_{{\rm{Resnik}}}},{\rm{BM}}{{\rm{A}}_{{\rm{Seco}}}}, simGI{C_{{\rm{Resnik}}}}$and $simGI{C_{Seco}}$) values across all species’ protein pairs in the protein family data sets.

**Figure 6.**
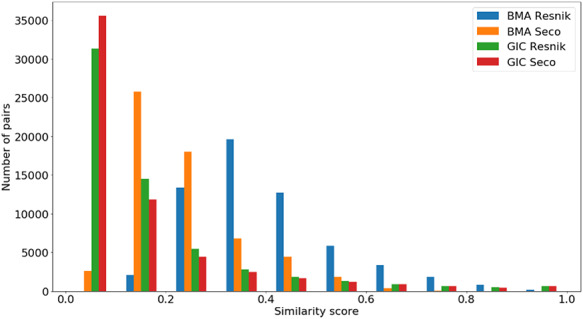
Distribution of all semantic similarity metrics ($BM{A_{Resnik}}$, $BM{A_{Seco}}$, $simGI{C_{Resnik}}$ and $simGI{C_{Seco}}$) values across all species’ protein pairs in the protein–protein interaction data sets.

Figures 4, 5 and 6 show the distribution of $si{m_{Pfam}}$ in the Protein Family data sets and semantic similarity in the Protein Family and PPI data sets, respectively. These distribution plots were designed using the data from the ‘One Aspect’, All species data set from the PPI and Protein Family data sets since they enclose all the pairs of proteins in their respective species-specific data sets of both annotation levels.

Figure 4 shows the $si{m_{Pfam}}$ score distribution for all the pairs of proteins in the Protein Family data sets. As expected, there is a balance between pairs of proteins with zero and total $si{m_{Pfam}}$. The partial $si{m_{Pfam}}$ pairs are more likely to have a similarity value between 0.1 and 0.5.

In Figure 5, we see that all similarity measures have a similar behaviour, except for $BM{A_{Resnik}}$. While the number of pairs grows as the value of $BM{A_{Resnik}}$ increases up until a similarity value of 0.4, this is not true for the other measures. With these, the number of pairs mostly decreases as similarity rises, with some exceptions in both ends of the similarity axis (e.g. the number of pairs of proteins rises when $simGIC$, for both ICs, equals 1).

A similar analysis can be done in Figure 6 for the same semantic similarity measures, where we see a distinct behaviour for $BM{A_{Resnik}}$, when compared to the other similarity measures. Even though the PPI data sets are balanced in terms of pairs of interacting/non-interacting proteins, and interacting pairs are expected to have higher semantic similarity, it is clear that their similarity values are skewed to lower values, with no raise in the number of pairs when the similarity reaches its maximum value.

Pearson correlation coefficient between all semantic similarity measures and the protein family proxies ($si{m_{Pfam}}$ and $si{m_{Seq}}$) was then assessed for the protein family data sets (Table 4), as well as between all semantic similarity measures and $si{m_{Seq}}$ and PPI for the PPI data sets (Table 5). Tables 4 and 5 present only correlation with $simGI{C_{Seco}}$, since it was the highest-scoring semantic similarity metric for most of the proxy measures, but the extended results are available online (https://github.com/liseda-lab/kgsim-benchmark). We found a positive correlation in all tests. In the PPI data sets, $BMA$ is shown to have a higher correlation with PPI, while $simGIC$ correlates better with $si{m_{Seq}}$, with both properties being IC independent. In the Protein Family data sets, once more, $simGIC$ correlates better with $si{m_{Seq}}$ than any $BMA$ approach and, although this is not true for all data sets, $BMA$ approaches show better correlation with $si{m_{Pfam}}$ than $simGIC$ (data available online at https://github.com/liseda-lab/kgsim-benchmark). In the Protein Family data sets, lower correlations were generally found for the ‘One aspect’ data sets, but this was not observed in the PPI data sets. In the PPI data sets, there is in most cases a lower correlation to sequence similarity, as expected (51), with the exception of the *H. sapiens* sets.

**Table 4.** Pearson correlation coefficient between semantic similarity (${\rm{simGI}}{{\rm{C}}_{Seco}}$ and protein family proxies ($si{m_{Seq}}$ and $si{m_{Pfam}}$) for all protein family data sets

	One aspect		All aspects	
Species	$si{m_{Seq}}$	$si{m_{Pfam}}$	$si{m_{Seq}}$	$si{m_{Pfam}}$
*D. melanogaster*	0.532	0.638	0.469	0.600
*E. coli*	0.386	0.455	0.421	0.450
*H. sapiens*	0.769	0.664	0.774	0.675
*S. cerevisiae*	0.642	0.568	0.636	0.549
*All*	0.592	0.616	0.619	0.605

**Table 5.** Pearson correlation coefficient between semantic similarity ($simGI{C_{Seco}}$) and sequence similarity ($si{m_{Seq}}$) and PPIs for all protein–protein interaction data sets

	One aspect		All aspect	
Species	$si{m_{Seq}}$	$PPI$	$si{m_{Seq}}$	$PPI$
*D. melanogaster*	0.488	0.719	0.516	im0.670
*E. coli*	0.229	0.624	0.181	0.604
*H. sapiens*	0.549	0.389	0.560	0.390
*S. cerevisiae*	0.300	0.563	0.353	0.538
*All*	0.375	0.473	0.440	0.445

### Genes-phenotypes benchmark data sets

**Figure 7.**
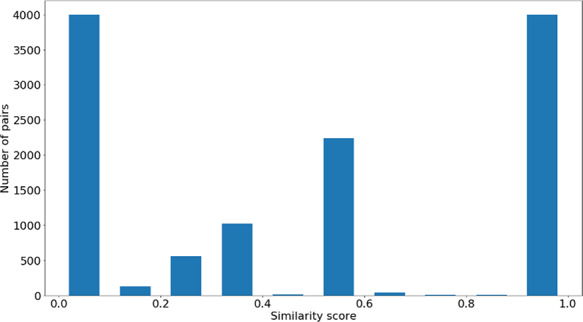
Distribution of $si{m_{PS}}$ values across all the pairs in the gene-phenotypes data sets.

**Figure 8.**
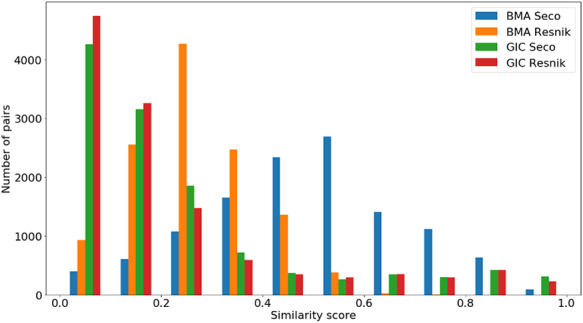
Distribution of all semantic similarity metrics ($BM{A_{Resnik}}$, $BM{A_{Seco}}$, $simGI{C_{Resnik}}$ and $simGI{C_{Seco}}$) values across all species’ protein pairs in the gene-phenotypes data sets.

The distribution of the $si{m_{PS}}$ values and semantic similarity values for this data set is shown in Figures 7 and 8. Figure 7 shows the $si{m_{PS}}$ score distribution for the pairs of genes in the gene-phenotypes data set. As expected, there is a balance between pairs of proteins with zero and total $si{m_{PS}}$. The partial $si{m_{PS}}$ pairs are more likely to have a similarity value between 0.1 and 0.5. In Figure 8, we see distinct behaviours for both $simGIC$ and $BMA$. While both $BMA$ approaches have a behaviour similar to that of a normal distribution, the $simGIC$ approaches have their values skewed to the left.

Pearson correlation coefficient between $si{m_{PS}}$ and all semantic similarity measures used are presented in Table 6, showing that all measures have a positive relation with $si{m_{PS}}$.

**Table 6.** Pearson correlation coefficient between $si{m_{PS}}$ and state of the art semantic similarity measures ($BM{A_{Resnik}}$, $BM{A_{Seco}}$, $simGI{C_{Resnik}}$ and $simGI{C_{Seco}}$) for the gene-pheno-types data set

		Pearson’s correlation
	*BMA_Resnik_*	0.572
	*BMA_Seco_*	0.590
	*simGIC_Resnik_*	0.478
	*simGIC_Seco_*	0.482

## Discussion

A big issue in the evaluation of semantic similarity measures is the diversity in the studies employed to do so. Semantic similarity measures are usually tested in a small and controlled set of data, developed for that study alone. This unsystematic assessment practice can lead to biases in the published results, especially if not compared with those of the state-of-the-art similarity measures in the same conditions, i.e. using the exact same version of the KG and the same entity pairs. Moreover, not employing a common strategy or, at least, the same data, makes the results from these studies not directly comparable across them.

This work aimed at tackling these issues by providing data sets with pairs of entities of different species, annotated with different ontologies and providing a combination of different similarity proxies and multiple state-of-the-art semantic similarity measures.

In order to guarantee that the semantic similarity measures can capture the functional similarity between the entities, their meaning must be well captured within the ontology context. This meant selecting entities annotated with more specific ontology classes (classes with fewer child classes), as sharing one, or more, of these classes will result in a higher and more significant semantic similarity between the two entities. This was done in order to tackle the shallow annotation problem for semantic similarity measures, which results in similarity values that are inconsistent with human perception due to shallowly described entities (11).

The definition of biological functional similarity is ambiguous because its exact meaning varies based on the context in which it is used (52). This bias is especially relevant when similarity is being defined by domain experts. For instance, let us imagine two protein kinases. These are proteins that modify other proteins by adding a phosphate group to them. A biochemist could deem the two proteins as very similar because they are both kinases; therefore, they have the same function. However, when analysing the two proteins from a physician’s perspective, they might be more interested in the role these two proteins play at the whole-organism level. The two kinases may be involved in different signalling pathways, and different mutations in these kinases might cause different diseases. Thus, from a physiological point of view, the two kinases are dissimilar. Not only is it unfeasible to ask domain experts to do this manual verification of similarity for every pair of biological entities there is, due to the amount of data in these domains, their perception will always be biased to their field of study or area of expertise.

The benchmark takes advantage of proxies of entity similarity for the evaluation of semantic similarity measures as a device for determining functional similarity of two biomedical entities. These measures of similarity, despite still capturing only one functional aspect of the entities at a time, bear two advantages: they rely on objective representations of the entities (e.g. gene sequence, protein structure, existence of PPIs, metabolic pathways affected by the disease) and calculate similarity using mathematical expressions or other algorithms Not only can these algorithms compare entities at a much faster rate than human experts, but they can also quantify the result from that comparison, as opposed to a similar/dissimilar assessment.

Out of the 21 data sets developed in this work, 20 are benchmark data sets for GO-based semantic similarity measures, because the GO is the most widely used ontology in the study of semantic similarity measures and its applications. The GO-based benchmark data sets can be divided by the similarity proxies employed in them. For each of the data sets, the combination of similarity proxies can be either (1) PPIs and sequence similarity or (2) protein family and sequence similarity. Sequence similarity is considered for both these data sets not only because it can be computed for any two proteins for which the protein sequence is known but also because sequence similarity does not show a strong enough relation with semantic similarity (47) to be used alone, as a sole evaluator of semantic similarity measures. As exposed before, PPIs and protein family are known to have different relation with the GO aspects. While protein family similarity is expected to correlate better with more matching classes from the Molecular Function subontology, the existence of a PPI is more likely to be in agreement with overlapping classes in the Cellular Component and Biological Process subontologies. Thus, a semantic similarity measure that has a positive relation with both these proxies is a semantic similarity measure that does a good job in capturing entity similarity as it is capable of considering different aspects of it.

The HPO-based data set considers only one similarity proxy, PS similarity. This similarity proxy can be seen as an evaluation of how well semantic similarity captures the probability of two genes being involved in the same disorders.

The diversity in the structure of the KGs and the similarity proxies selected for the construction of these data sets suggests that testing the same semantic similarity in differently targeted data sets can be a good evaluator of its ability to generalize to different KGs, entity types and applications.

Furthermore, the data sets follow the guidelines for quality benchmark data sets, namely relevance, representativeness, non-redundancy, scalability and reusability. Even though benchmark data sets should be non-redundant, the overlap between data sets of the same species, but at a different level of annotation completion, can be used to evaluate the impact of more thoroughly described proteins in the performance of semantic similarity measures. The ‘All’ data sets in each level of annotation completion are a compilation of all the protein pairs in each of the species-specific data sets. Even though, once more, there is redundancy between these and the species-specific data sets, the ‘All’ data sets are far larger and can be used for a comparative evaluation of the scalability of the semantic similarity measures or semantic similarity-based approaches. Representativeness was a feature of special importance when designing these data sets, for instance, upon selecting pairs of proteins based on their protein family similarity, because the evaluation of semantic similarity measures should be done in both similar and dissimilar pairs of entities. Additionally, should these data sets be used for supervised learning applications, these predictors will benefit from learning from a more general data set. If the cases used for training are particularly biased towards a single feature, the performance of the predictor will be biased as well.

## Usage notes

All data sets are available online (https://github.com/liseda-lab/kgsim-benchmark) with a CC BY 4.0 license. In addition, we make available all the data that was used to compute the semantic similarity measures in two different formats (1) Separate files for the ontologies (OBO or OWL format) and the annotations for each species to make up the KG, and (2) KGs, in OWL format, containing both the ontology and the annotations of each species. This allows for a direct comparison with the pre-computed semantic similarity measures, as well as facilitates the direct comparison between different works, without needing to implement and/or compute the results.

The benchmark supports simple evaluation metrics, such as computing Pearson’s correlation coefficient, but it also supports more complex evaluations. For instance, the PPI data sets also support prediction of PPIs based on semantic similarity (48).

The steps to perform the benchmark evaluation for a new KG-based semantic similarity measure are as follows:

Select the benchmark data sets that will be used, download them and the associated KG data sets;
Using the novel measure, calculate the similarity for all entity pairs in the benchmark data sets using the benchmark KG;
Compute evaluation metrics against proxy similarity values and representative semantic similarity scores;
Upload the novel semantic similarity results to a data-sharing platform to support future direct comparisons.

## Concluding remarks

The collection of benchmark data sets we present aims at supporting the large-scale evaluation of KG-based semantic similarity based on four different similarity proxies: protein sequence similarity, existence of PPIs, protein family similarity and PS similarity. The first three proxies represent protein similarity and can be used to evaluate GO-based semantic similarity measures, whereas the latter is a proxy for gene similarity for the evaluation of HPO-based semantic similarity measures.

All data sets and KG data used to compute semantic similarity are available online. This allows for a direct comparison with the pre-computed semantic similarity measures, as well as facilitates the direct comparison between different works using this resource. For this reason, the benchmark will purposefully remain static for a few years, following the approach used by CESSM (33), released in 2009 and updated in 2014. Parallel updates to the benchmark data sets will include new KGs, with updated attributes for entity selection and new similarity proxies.

The benchmark supports simple evaluation metri-cs, such as computing Pearson’s correlation coefficient between the semantic similarity measures and the similarity proxies, but it also supports more complex evaluations. For instance, the PPI data sets also support prediction of PPIs based on semantic similarity, as done in Sousa *et al*. (48). Despite being domain-specific, we expect this collection to be useful beyond the biomedical domain. Similarity computation within a KG is a fundamental building block of many semantic web applications ranging from data integration to data mining, meaning the benchmark data sets can be used for the evaluation of semantic similarity measures developed outside the biomedical domain.

Additionally, the general approach developed for the creation of the data sets is generalizable to any domain where a similarity proxy can be created, making the development of analogous benchmark data sets outside the biomedical domain a possibility.
